# Endothelial and cancer cells interact with mesenchymal stem cells *via* both microparticles and secreted factors

**DOI:** 10.1111/jcmm.12391

**Published:** 2014-09-23

**Authors:** Thomas P Lozito, Rocky S Tuan

**Affiliations:** Department of Orthopaedic Surgery, Center for Cellular and Molecular Engineering, University of Pittsburgh School of MedicinePittsburgh, PA, USA

**Keywords:** mesenchymal stem cells (MSCs), microparticles (MP), extracellular vesicles, endothelial cells (ECs), perivascular niche

## Abstract

Tightly associated with blood vessels in their perivascular niche, human mesenchymal stem cells (MSCs) closely interact with endothelial cells (ECs). MSCs also home to tumours and interact with cancer cells (CCs). Microparticles (MPs) are cell-derived vesicles released into the extracellular environment along with secreted factors. MPs are capable of intercellular signalling and, as biomolecular shuttles, transfer proteins and RNA from one cell to another. Here, we characterize interactions among ECs, CCs and MSCs via MPs and secreted factors *in vitro*. MPs and non-MP secreted factors (Sup) were isolated from serum-free medium conditioned by human microvascular ECs (HMEC-1) or by the CC line HT1080. Fluorescently labelled MPs were prepared from cells treated with membrane dyes, and cytosolic GFP-containing MPs were isolated from cells transduced with CMV-GFP lentivirus. MSCs were treated with MPs, Sup, or vehicle controls, and analysed for MP uptake, proliferation, migration, activation of intracellular signalling pathways and cytokine release. Fluorescently labelled MPs fused with MSCs, transferring the fluorescent dyes to the MSC surface. GFP was transferred to and retained in MSCs incubated with GFP-MPs, but not free GFP. Thus, only MP-associated cellular proteins were taken up and retained by MSCs, suggesting that MP biomolecules, but not secreted factors, are shuttled to MSCs. MP and Sup treatment significantly increased MSC proliferation, migration, and MMP-1, MMP-3, CCL-2/MCP-1 and IL-6 secretion compared with vehicle controls. MSCs treated with Sup and MPs also exhibited activated NF-κB signalling. Taken together, these results suggest that MPs act to regulate MSC functions through several mechanisms.

## Introduction

Mesenchymal stem cells (MSCs) have long attracted attention because of their differentiation capabilities, yet their most interesting behaviours may involve their interactions with other cells. For example, there is strong evidence to suggest that MSCs occupy a perivascular niche in a variety of vascularized tissues [1–5]. In fact, MSCs have been described as a type of pericyte, a microvascular cell type analogous to the smooth muscle cells of macrovessels [5,6]. MSCs express pericyte markers [1,2], and exhibit pericyte activities, such as enhancing vessel formation and stabilization through paracrine interactions [3,4]. Furthermore, both MSCs and pericytes display similar differentiation capabilities [5–8]. As pericytes, wrapped around blood vessels in their perivascular niches, MSCs closely interact with endothelial cells (ECs), the primary vascular cell type that makes up the walls of blood vessels. MSCs are also able to leave their perivascular niche during wound healing and disease conditions. For example, MSCs home to tumours, where they interact with both cancer cells (CCs) and ECs [9,10].

Mesenchymal stem cells naturally interact with ECs and CCs, and vice versa, and studies involving crosstalk between MSCs and these cell types have traditionally been broken down into 3 main components: (*i*) cell–cell [11], (*ii*) cell–matrix [11,12] and (*iii*) cell-soluble factor interactions [13]. One of the most interesting and relatively under-studied areas of study involving interactions between cells and their environment concerns extracellular membrane vesicles known as microparticles/microvesicles (MPs). MPs are released into the extracellular environment by a variety of cell types [14–21] and are divided into two groups, exosomes and ectosomes, based on size (50–1000 nm in diameter for ectosomes [14,22], 50–100 nm for exosomes [23]), composition and origin. Exosomes are enriched in tetraspanins, milk fat globule-EGF factor 8 (MFG-E8) and MHC class II molecules [23], while ectosomes are associated with their own set of markers, including MMPs [24,25]. Ectosomes are formed directly by ectocytosis [22,24,26], while exosomes originate from multivesicular bodies (MVBs) that result when endosomes bud inwards into their lumens [27–29]. Exosomes are released as MVBs fuse with the plasma membrane and release their intraluminal vesicles. Both exosomes and ectosomes are distinct from apoptotic bodies, which are larger (1–4 μm), formed at the end of apoptosis, and are usually immediately taken up by macrophages [30,31].

Interestingly, MPs contain membrane and cytosolic components that can be transferred from one cell to another as the particles are released and fuse with neighbouring cells. For example, exosomes released by human mast cell lines are capable of transferring mRNAs and microRNAs to other mast cells. Once inside the recipient cell, this ‘exosomal shuttle RNA’ is functional and affects cell behaviour [32]. MPs also contain a wide range of membrane and cytosolic proteins [33,34] that can also be shuttled between cells. MPs have been shown to mediate the intercellular transfer of epidermal growth factor receptor [35,36], tissue transglutaminase [37] and chemokine receptor 5 [38]. In all these cases, the recipient cell exhibited altered behaviour in response to the transfer of biomolecules via MPs.

Microparticles also signal to recipient cells independent of biomolecular transfer by acting as circulating signalling complexes. Indeed, MPs have been shown to present bioactive signalling molecules of their surfaces, including Wnt [39] and Hedgehog ligands [40,41]. MPs also contain internalized growth factors, such as VEGF [42], fibroblast growth factor-2 (FGF-2) [43] and interleukin-1β (IL-1β) [44], which are released upon degradation. These membrane-associated growth factors bind receptors on the receiving cells and activate various cell behaviours, including migration [39], cytokine release [45,46], prostaglandin production [47], MMP expression [46] and signalling pathway activation [45].

As stem cells multipotent for mesenchymal lineages, MSCs are profoundly influenced by signals originating from their local environments, signals that derive, in part, from neighbouring cells. ECs and CCs, both of which are included in the long list of cell types that release MPs [19,20,25] and, by virtue of their physical proximity, probably interact closely with MSCs. This study considers whether interactions among ECs, CCs and MSCs include MP-mediated signalling and how MP signalling influences the functional activities of MSCs.

## Materials and methods

### Cell culture

Human MSCs were isolated from hip replacement surgical waste collected from three separate patients according to previously published protocols [48] with Institutional Review Board approval. Briefly, femur head bone marrow was repeatedly flushed with DMEM by using a 25-gauge syringe. The medium was collected and passed through a 70 μm mesh filter. The cells were pelleted by centrifugation at 200 × g for 5 min and then resuspended in growth media [DMEM supplemented with 10% lot-selected foetal bovine serum (FBS) + 1% penicillin and streptomycin (PS)] and plated in tissue culture flasks. After 4 days, fresh medium was added 1:1. After another 4 days, cells were trypsinized and passaged. The resultant cells were referred to as passage 1. MSCs were used in experiments at passage 3, which were repeated as 3 experimental replicates with two patients each. Isolated MSC populations were validated with flow cytometry analysis of cell surface marker expression. MSCs were trypsinized, and cell suspensions were washed in PBS/1% FBS, blocked in 10% mouse serum, probed with PE-conjugated isotype controls IgG1,κ and IgG2a,κ) or PE-conjugated primary antibodies (anti-CD73, CD90, CD105, CD45, CD19, CD34 and CD14), fixed with 4% paraformaldehyde and analysed with a BD FACSAria II for PE (488λ laser, 575/25BP filter, Blue-2 detector) fluorescence. All isotype controls and PE-conjugated antibodies were purchased from BD Biosciences (San Jose, CA, USA). MSCs were positive for CD73, CD90 and CD105 and negative for hematopoietic markers CD45, CD19, CD34 and CD14 (Fig. S1).

The human dermal microvascular EC line HMEC-1 was maintained in the EC medium EGM-2-MV (Lonza, Walkersville, MD, USA), consisting of a basal media supplemented with FBS (5%), human epidermal growth factor (hEGF) (10 ng/ml), hydrocortisone (1 μg/ml), gentamicin (50 μg/ml)/amphotericin-B (50 ng/ml; GA), human recombinant fibroblast growth factor-beta (hFGF-b), VEGF, insulin-like growth factor (R3-IGF-1) and ascorbic acid (the concentrations of hFGF-b, VEGF, R3-IGF-1 and ascorbic acid included as part of the EGM-2-MV medium kit are not disclosed by the manufacturer). The human fibrosarcoma CC line HT1080 was maintained in α-MEM supplemented with 10% FBS and 1% PS.

### MP isolation

EC and CC cultures to be used to generate MPs were washed with PBS and cultured in serum-free (SF) Phenol Red-free (PF) EC medium containing hEGF, hydrocortisone, hFGF-b, VEGF, R3-IGF-1, ascorbic acid, and GS and supplemented with Insulin-Transferrin-Selenium for 24 hrs. [Note: We have found that the addition of these supplements is essential for maintaining EC viability over 24 hrs of serum starvation and, subsequently, avoiding contamination of harvested MPs with apoptotic bodies (Fig. S2)]. Cell-conditioned medium (CM) was cleared of cells and debris through sequential centrifugation at 600 × g for 15 min and then at 1500 × g for 15 min and ultracentrifuged for 2 hrs at 100,000 × g at 4°C using a Beckman XL-70 Ultracentrifuge (SW40Ti rotor). The ultracentrifugation pellets, referred to here as MPs, were resuspended in either SF PF medium M199 (Invitrogen) or protein collection buffer (Millipore) for analysing by Western blot. The non-pelleted supernatants, referred to here as Sup, were collected and concentrated via 3 kD MWCO spin concentrators (Millipore). MP and Sup aliquots were analysed by BCA for protein concentration.

The protocol described above was used to isolate MPs for experiments except for two exceptions. The first involved EC MP characterization experiments, where a differential centrifugation protocol was employed to yield ectosome and exosome vesicle fractions of total MP populations [19,49]. EC-conditioned media was spun at 300 × g for 15 min followed by 2000 × g for 15 min to clear cells and debris, and then spun at 10,000 × g for 30 min at 4°C to pellet ectosomes, which were resuspended in either medium M199 or protein collection buffer. The supernatant was centrifuged at 100,000 × g for 1 hr at 4°C to pellet exosomes, which were again collected in either M199 medium or protein collection buffer.

The second exception involved experiments validating GFP localization to both MP and Sup fractions in samples collected from ECs transduced with lentiviral CMV-GFP constructs. ExoQuick-TC™ kits (System Biosciences, Mountain View, CO, USA) were used to isolate microvesicle and Sup samples according to the manufacturer's instructions.

### Electron microscopy

Ultracentrifugation pellets resuspended in PBS were applied to collodion-coated grids, negatively stained with 1% phosphotungstic acid, pH 7.0 and observed with transmission electron microscopy (JEM-1011 Electron Microscope; JEOL USA, Peabody, MA, USA). Sizing of MPs were carried out by measuring the dimensions of 250 randomly selected MPs in the electron micrographs, and the data were analysed with NIH ImageJ using the Particle Analysis function.

### CMV-GFP lentiviral transduction

Endothelial cell cultures were washed with PBS and treated with a custom-made lentiviral CMV-GFP construct [50] (vector background similar to Invitrogen CMV-GFP construct) diluted with EGM-2 MV media without antibiotics, washed with PBS and fed full EGM-2 MV media. The transduced EC cultures were cultured for an additional 48 hrs, washed with PBS, and MP and Sup samples were collected as described above. MSC cultures were then treated with MPs collected from either transduced or untransduced ECs as described below.

### Western blot

Microparticle and cellular protein samples were collected in Total Protein Collection (TPC) buffer (Millipore). MP pellets were resuspended in TPC buffer (20 μl per ultracentrifuge tube) following ultracentrifugation. To collect cellular proteins, cultures were washed with ice-cold PBS and lysed with TPC buffer (10 μl/cm^2^). Protein samples were subjected to reducing SDS-PAGE, transferred to polyvinylidene fluoride membranes, probed with primary antibodies [anti-catalase, calnexin, HDAC1, Rab5, caveolin, ERAB, GAPDH (EMD Millipore), LAMP-2, CD63 (Systems Biosciences), HSP70 (Systems Biosciences), GFP, NF-κB p50 (EMD Millipore), MMP-1, MMP-3, CCL-2, IL-6 and MMP-2], incubated with horseradish peroxidase (HRP)-conjugated secondary antibodies, incubated with chemiluminescent HRP substrate and imaged with a Fotodyne/Analyst FX CCD camera system. All primary antibodies were purchased from Abcam unless otherwise specified. Densitometric analyses of Western blots were performed with ImageJ.

### Cell viability and proliferation

Endothelial cell viability and MSC proliferation were measured with the CellTiter 96® AQueous One Solution Cell Proliferation Assay (MTS; Promega, Fitchburg, WI, USA) according to the manufacturer's instructions. In reporting MSC proliferation results, 490 nm absorbances were related to cell numbers using standard curves.

### Real-time RT-PCR

RNA was collected from cells treated with MPs by using the RNeasy® Plus Mini Kit (Qiagen, Hilden, Germany) according to the manufacturer's instructions. Cell cultures were trypsinized and washed extensively prior to RNA isolation. The RT^2^ First Strand Kit (Qiagen) was used to convert mRNA into cDNA according to the manufacturer's instructions. Samples were analysed via Signal Transduction PathwayFinder PCR Arrays (Qiagen).

### MP–MSC interaction studies

Cell monolayer cultures were grown to 95% confluency in their respective growth media. Growth medium was then removed, and the cultures were washed with PBS. MP or Sup was added at identical concentrations (typically 25 μg/ml). MP vehicle control (MVC) in the form of SF PF medium M199 or Sup vehicle control (SVC) in the form of SF PF EC medium was added at identical volumes to MP and Sup samples, respectively. Cells were cultured in treatments for 0, 4, 24, 48 or 72 hrs. The respective culture medium samples were collected from cells treated with MVC, MP, SVC or Sup, centrifuged at 1050 × g for 10 min to remove cell debris and stored at −80°C. Before collection, cell samples were washed 5 times in M199 and trypsinized for 5 min at 37°C. Defined trypsin inhibitor (Invitrogen) was added and the cell suspensions were centrifuged at 200 × g for 5 min. Cell pellets were washed with M199 medium and harvested for protein or mRNA. Cell-free controls (CFCs) consisted of identically treated tissue culture plastic. For isolation of cytoplasmic and nuclear proteins, MP-treated cells were processed with the NE-PER Nuclear Protein Extraction Kit (Thermo Fisher Scientific, Rockford, IL, USA) according to the manufacturer's instructions.

### Migration studies

Migration studies were performed by using the Roche xCELLigence System (Roche Diagnostics, Indianapolis, IN, USA). The system consists of plates of modified Boyden chambers, with upper and lower chambers separated by a cell-permeable membrane lined with electrodes. Cells are seeded into the top chambers, and candidate chemoattractants are placed in the bottom chambers. As cells migrate across the membrane, they contact electrodes, which the system reads as migrating cells. MVC, MP, SVC and Sup were added to the bottom chambers. MP and Sup samples were added at 100 μg/ml, and MVC and SCV samples were added at identical volumes to MP and Sup samples, respectively. Cells (40,000 per well) were added to top wells, and the plates were scanned every 15 min for 24 hrs.

### Live cell imaging

Endothelial cell cultures were incubated with CellMask™ Orange (Invitrogen; 7 μg/ml) for 5 min at 37°C, washed extensively with PBS, and fluorescently labelled MPs were collected as described above. Labelled MP solution was diluted 1:100 in SF PF medium and administered to MSCs cultured on Mattek Dishes. MSC/MP cultures were analysed with a custom-built live cell imaging system based on the Nikon sweptfield confocal microscope. Images of the MSC/MP cultures were taken every minute for 2 hrs, beginning at the time of MP-administration.

### Flow cytometry quantification of biomolecular transfer from EC MPs to MSCs

Confluent EC cultures were incubated with DiD (Invitrogen) diluted 1:200 in SF PF medium for 25 min at 37°C, washed extensively with PBS, and DiD-labelled MPs were collected and used to treat MSCs as described above. MP-treated MSCs were washed 5 times in M199 medium, collected by trypsinization, fixed with 4% paraformaldehyde, incubated with 5 mM DAPI for 10 min, resuspended in PBS/1% FBS and analysed with a BD FACSAria II for DAPI (405λ laser, 450/50BP filter, Vi-1 detector), GFP (488λ laser, 530/30BP filter, Blue-1 detector) and DiD (640λ laser, 670/30BP filter, Red-1 detector) fluorescence.

### Statistics

Each sample was analysed over 3–5 experimental replicates. Quantified results are expressed as the mean ± SD, and significant differences among experimental conditions were determined by two-tailed Student's *t*-tests for two-group comparisons or anova for multiple group comparisons. Significance was considered at p < 0.05. Experiments involving MSCs were repeated with cells isolated from at least 2 patients. Results from the same representative patient are presented.

## Results

### The EC MPs used in this study exhibit characteristics of ectosomes

**Fig. 1:**
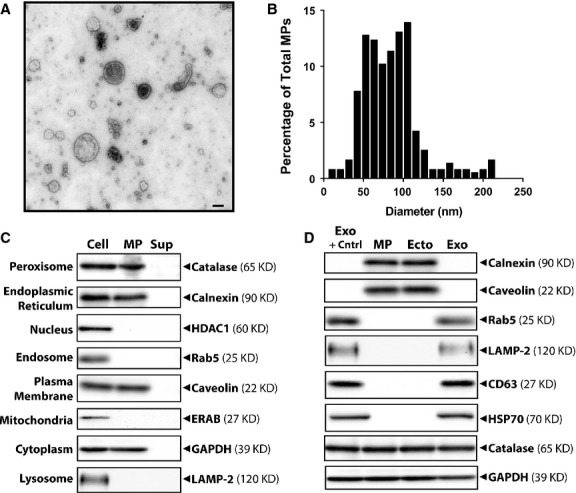
Endothelial cell (EC) microparticle (MP) validation and characterization. (**A**) Transmission electron micrograph of EC MPs. (**B**) Size profile of EC MPs. (**C**) Western blot analysis of organelle markers among protein samples (10 μg total protein) collected from parent ECs (Cell) and MP and Sup media fractions. (**D**) Western blot analysis of ectosomal and ectosomal markers among protein samples (5 μg total protein) collected from unfractioned MPs, and ectosome (Ecto) and exosome (Exo) fractions isolated using differential centrifugation. A commercially available exosome positive control (Exo + Cntrl) was also analysed.

Endothelial cell MPs were observed by negative staining and transmission electron microscopy as intact membranous structures (Fig. 1A). The majority of isolated EC MPs (71%) were between 50 and 115 nm in diameter (Fig. 1B), and, therefore, within the size ranges of both ectosomes and exosomes, but not apoptotic bodies [31,51]. To aid in making this distinction, EC MPs were analysed for the presence of various organelle markers (Fig. 1C). Ectosomes, which originate from the plasma membrane, are reported to include membrane, endoplasmic reticulum (ER) and cytoplasmic components [49], while exosomes, originating from intracellular MVBs, are reported to include endosomal and lysosomal markers [34,49,52]. EC MPs were also analysed for nuclear and mitochondrial markers, which are expressed by apoptotic bodies [51]. The EC MPs isolated as part of this study were positive for the ER marker calnexin, the plasma membrane marker caveolin and the cytoplasmic marker GAPDH. The EC MPs were negative for the nuclear maker HDAC1, the endosomal marker RAB5, the mitochondrial marker ERAB and the lysosomal maker LAMP-2. These results suggest that the EC MPs used in this study were ectosomes and not exosomes or apoptotic bodies. Interestingly, the EC MPs were positive for the peroxisomal marker catalase, which is in agreement with previous reports that describe catalase expression by ectosomes [53]. All of the markers tested were expressed by the parent ECs, while none of these markers was detected in Sup samples.

While the vast majority of the EC MPs used in this study exhibit ectosome characteristics, our isolation procedure (*i.e*. single-step ultracentrifugation), undoubtedly results in a heterogeneous vesicle population that possibly includes exosomes. Thus, we used a differential centrifugation vesicle preparatory procedure to further characterize EC MPs by fractionating the MP vesicle population into ectosomes and exosomes. All three populations (unfractioned MP, ectosomes, and exosomes) were analysed along with a commercially available exosome positive control (Systems Biosciences) via Western blot for various ectosomal and exosomal markers (Fig. 1D). The same amount of total protein (5 μg) was analysed of each sample, so vesicle population underrepresented in unfractionated MP preparations would be enriched in fractioned preparations of exosomes and ectosomes. Unfractioned MP and ectosome vesicle populations were positive for the ectosomal markers calnexin and caveolin, and negative for the exosomal markers Rab5, LAMP-2, CD63 and HSP70, while the exosome fraction and positive control expressed exosomal, but not ectosomal, markers. All samples tested were positive for the peroxisomal marker catalase and the cytoplasmic marker GAPDH. We also tested the effects of ectosome and exosome vesicle fractions on MSCs, but did not detect significant differences in vesicle-induced cell migration or proliferation (Fig. S3). Thus, these results suggest that the vast majority of the vesicles used in this study can be classified as ectosomes. As both ectosomes and exosomes performed comparatively in MSC-based assays, and given that exosomes make up a very small fraction of EC MP vesicle population and that exosomes are significantly more costly in terms of both time and supplies to prepare, all further experiments described in this study were performed by using unfractioned MP vesicle populations.

### MPs transfer cellular components from ECs to MSCs

**Fig. 2:**
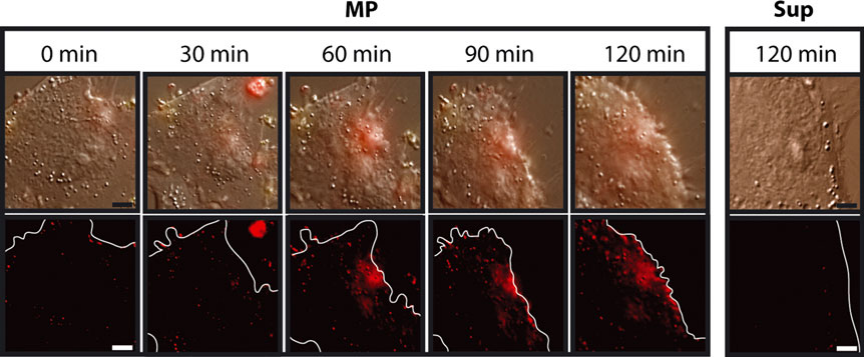
Live cell imaging of mesenchymal stem cells (MSCs) interacting with fluorescently labelled endothelial cell (EC) microparticles (MPs). An MSC was tracked during its interactions with CellMask™ Orange I-labelled EC MPs over 120 min. As a negative control, an MSC that had been incubated for 120 min with Sup collected from fluorescently labelled ECs is presented. Images displayed in the top row are combinations of bright field and fluorescence (Ex 488/Em 575), while images in the bottom row are fluorescence only. Cell outlines are depicted in bottom row images by a solid white line; bar = 1 μm.

To demonstrate MP interaction and fusion of EC MPs and MSCs, live cell imaging was used to observe MSC cultures incubated with fluorescently labelled EC MPs. MP and Sup were isolated from ECs labelled with the membrane dye CellMask™ Orange. Labelled MPs were clearly observed as they descended onto and bound unlabelled MSCs (Fig. 2). Upon binding, the MPs fused with the MSCs, transferring their fluorescent membrane-associated dye to the MSC surfaces over the 120 min assayed. These fluorescent membrane patches localized with the point of contact between an MP and the MSC surface. Serving as negative controls, MSCs incubated with Sup samples collected from fluorescently labelled MSCs did not exhibit fluorescent membrane patches over the time period assayed. These results suggest that EC MPs bind and fuse with the cell membranes of MSCs, and that EC components, in this case membrane dye, are effectively transferred to MSCs via MPs, but not non-MP secreted factors (Sup).

**Fig. 3:**
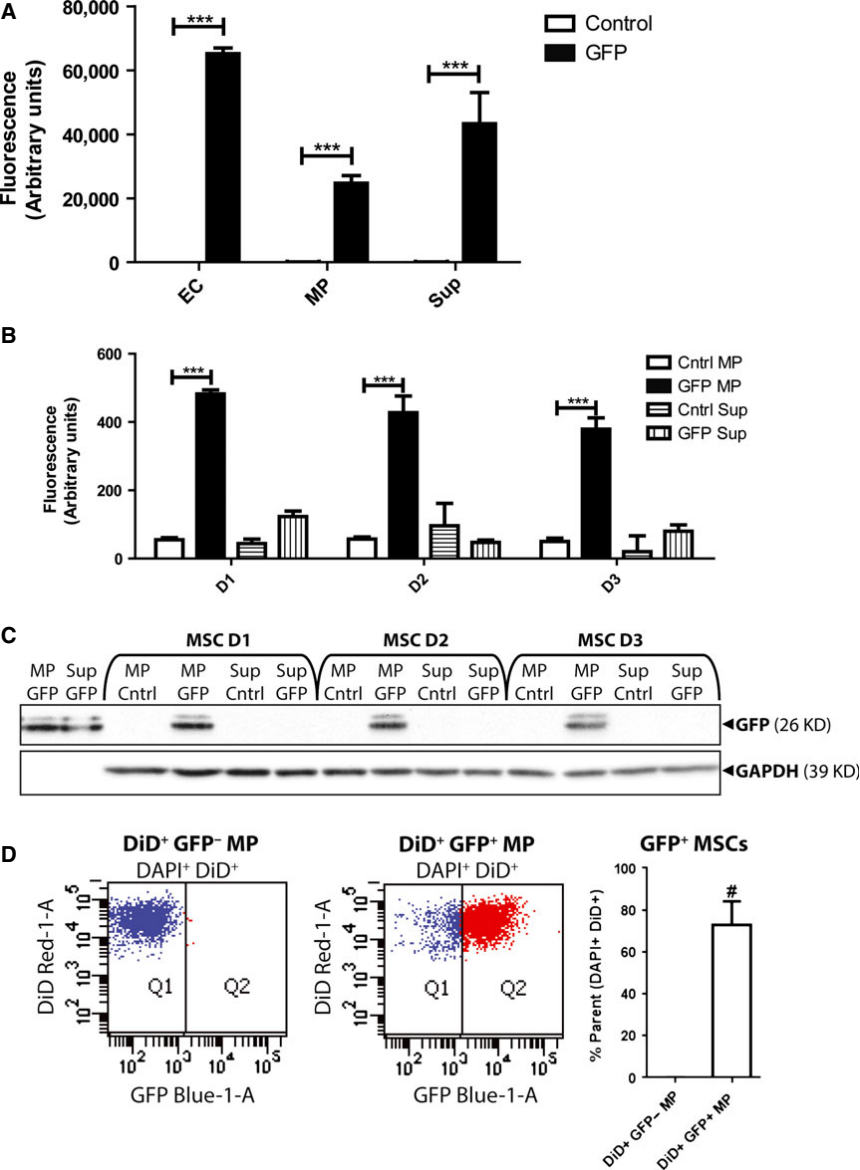
Microparticles (MPs) transfer biomolecules from endothelial cells (ECs) to mesenchymal stem cells (MSCs). (**A**) Fluorescence spectroscopy analysis of whole cell (EC), MP, and Sup protein samples (20 μg) collected from ECs transduced with (GFP) or without (Control) CMV-GFP lentivirus. (**B**) Fluorescence spectroscopy analysis of cell lysates (20 μg) collected from MSCs incubated with MP or Sup (25 μg protein/ml) collected from untransduced (control) or GFP-transduced (GFP) ECs for 1, 2 or 3 days. ***, p < 0.001. (**C**) Western blot analysis of GFP contents of lysates (20 μg) collected from MSCs incubated with MP or Sup proteins collected from GFP or control ECs for 1, 2, or 3 days. (**D**) Flow cytometry analysis of MSCs treated with DiD+ GFP- (gating control) or DiD+ GFP+EC MPs for 24 hrs. Q1 and Q2 denote DiD+ GFP-and DiD+GFP+ MSC populations, respectively. Quantification of these MSCs from 3 independent experiments is presented in the histogram on the right. #, p < 0.001.

These experiments were next performed with EC MPs engineered to contain cytosolic GFP. EC MP and ultracentrifugation supernatant (Sup) samples were collected from EC cultures transduced with a lentiviral CMV-GFP construct. Samples collected from transduced cultures contained significantly higher fluorescence measurements than untransduced controls for all fractions (Fig. 3A). Thus, GFP was transferred from the ECs to the MPs, but also to the Sup fraction. (Note: To rule out the possibility that GFP inclusion in Sup samples was because of incomplete MP versus Sup fractionation, and vice versa, MP and Sup samples collected from transduced ECs were also isolated by an alternative approach by using ExoQuick-TC™ kits. GFP was detected by Western blot in all samples regardless of isolation protocol (Fig. S4), confirming that GFP is included in both MP and Sup media fractions). MSCs were treated with MP and Sup GFP (25 μg protein/ml). After 1, 2 or 3 days, MSC cell lysates were collected and analysed (20 μg protein per sample) via fluorescence spectroscopy (Ex 488/Em 525) (Fig. 3B). Only lysates collected from MSCs treated with GFP-MP exhibited significantly enhanced fluorescence compared with vehicle controls (fractions collected from untransduced ECs). MP-transferred GFP was retained for at least 3 days. These results were confirmed with Western blot (Fig. 3C), as GFP was detected only in MSCs treated with GFP-MPs, indicating that only MP-associated GFP was transferred to MSCs. Again, this transference was retained for at least 3 days. (Note: Neither GFP mRNA nor lentiviral RNA/protein was ever detected in MSCs incubated with MPs isolated from transduced cells (data not shown), suggesting that the GFP detected in MSCs results from protein transfer from MPs to MSCs.) Thus, only MP-associated proteins were taken up and retained by MSCs, suggesting a level of cellular specificity in which MP biomolecules, but not secreted factors, are shuttled between MSCs and ECs.

The above experiments were combined to yield a new quantitative method to evaluate MP-cell interactions and biomolecular transfer. EC cultures were transduced with lentiviral CMV-GFP and labelled with the fluorescent membrane dye DiD, yielding EC MPs co-labelled with DiD and GFP. MSCs were incubated with DiD^+^ GFP^+^ MPs (100 μg protein/ml) for 24 hrs, stained with DAPI and analysed via flow cytometry (Fig. 3D). Selecting for DAPI^+^ populations ensured that MSCs, not MPs, were included in analysis. Having shown that lipophilic fluorescent dyes are quickly and efficiently transferred from labelled MPs to MSCs during MP-MSC interactions, DiD^+^ populations were selected to concentrate analysis on MSCs that have interacted with MPs. DAPI^+^ DID^+^ populations were analysed for GFP fluorescence by using MSCs treated with DiD^+^ GFP^−^ MPs to set gating strategies for GFP fluorescence. Of the MSCs treated with DiD^+^ GFP^+^ MPs, clear populations of DiD^+^ GFP^+^ MSCs were detected, representing cells that had taken up and retained both DiD and GFP from MPs. Thus, MSCs populations that received both membrane and cytosolic proteins from MPs were identified.

### MPs induce MSC migration and proliferation

**Fig. 4:**
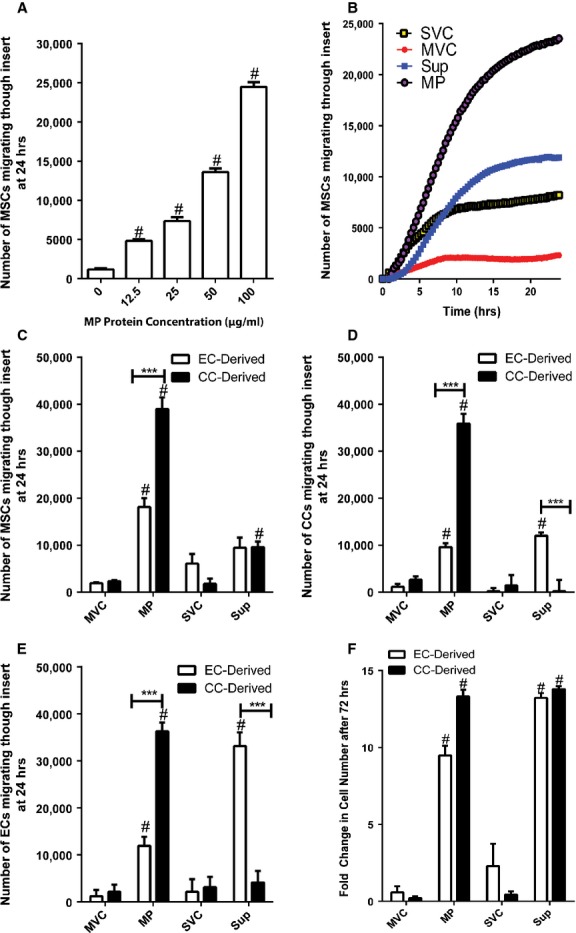
Microparticles (MPs) induce mesenchymal stem cell (MSC) migration and proliferation. (**A**) MSC chemotaxis in response to endothelial cell (EC) MPs (0–100 μg protein/ml) after 24 hrs. #, p < 0.001, compared with vehicle control. (**B**) Representative real-time migration assay plot of MSC chemotaxis towards EC MVC, MP, SVC, or Sup (100 μg protein/ml) over 24 hrs. (**C**) MSC, (**D**) CC, (**E**) or EC chemotaxis towards EC- or CC-derived MPs and Sup (100 μg protein/ml) after 24 hrs. #, p < 0.001, compared with vehicle control; ***, p < 0.001. (**F**) Effects of treatment (72 hrs) with MP and Sup (100 μg protein/ml) collected from ECs or CCs on MSC proliferation. Fold changes of 0 represent no changes in cell numbers compared with those at the start of the experiments. #, p < 0.001, compared with vehicle control.

Mesenchymal stem-cell migration in response to MPs was measured by using the Roche xCELLigence System consisting of Transwells with electrodes coating one side of the cell-permeable membrane. Chemoattractant placed in the bottom chamber induces migration in cells placed in the top, and the system measures cells as they crawl through the membrane and contact the electrodes. This allows for very sensitive detection of migrating cells in real time. The system was used to measure the MSC migration dose response to EC MPs (0–100 μg protein/ml) (Fig. 4A). The range tested induced a direct response in MSC migration, indicating that MSC migration towards EC MPs is concentration-dependent. Figure 4B shows representative plots of migration in response to EC MVC, MP, SVC or Sup (100 μg protein/ml) over 24 hrs. Both EC MPs and Sup significantly induced chemotaxis in MSCs compared with vehicle controls. EC MPs were significantly more chemoattractive to MSCs than EC Sup.

Mesenchymal stem cells naturally home to tumours, where they interact with CCs [9,10]. Furthermore, we sought to include a cell-type control to determine whether the effects observed with EC MPs were specific for epithelial-derived vesicles or common for MPs regardless of parent cell identity. Thus, MPs derived from the mesenchymal CC line HT1080 were included in this study, and MSC migration induced by either EC- or CC-derived MPs were compared (Fig. 4C). (Note: CC MPs exhibited similar expression of organelle markers when compared with EC MPs (Fig. S5), suggesting that both sets of MPs are produced via similar mechanisms and predominantly consist of ectosomes.) CC MPs were significantly more chemoattractive to MSCs than EC MPs, and only CC Sup was significantly chemoattractive to MSCs compared with vehicle controls. Thus, the identity of the parent cell type clearly affects the degree to which MPs induce MSCs to migrate. These experiments were expanded to test the effects of EC- and CC-derived MPs on CCs and ECs to determine if MP effects on migration differed with the receiving cells. As in MSCs, CC MPs induced more migration in ECs and CCs than EC MPs (Fig. 4D and E). Interestingly, EC-derived Sup, but not CC Sup, induced migration in CCs and ECs.

Finally, the effects of CC and EC MPs on MSC proliferation were considered. Identical numbers of MSCs were plated and treated with EC- or CC-derived MPs or Sup under SF conditions for 0 or 72 hrs and analysed for proliferation by using MTS assays (Fig. 4F). Both EC-and CC-derived MPs and Sup significantly enhanced MSC proliferation compared with vehicle controls.

### MPs activate MSC cytokine secretion

**Fig. 5:**
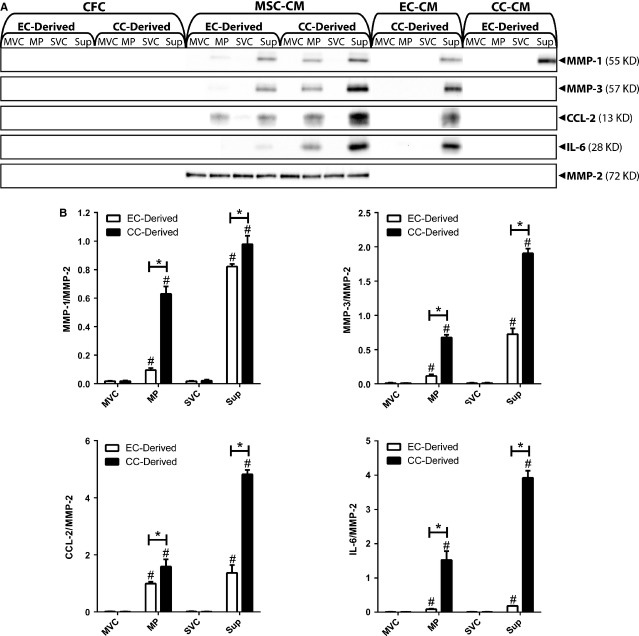
Microparticles (MPs) and Sup induce mesenchymal stem cell (MSC) cytokine secretion. (**A**) Western blot analysis of MSC-conditioned medium (CM), endothelial cell-cell-conditioned medium (EC-CM), or CC-CM collected from cells treated with EC- or CC-derived MVC, MP, SVC, or Sup (25 μg protein/ml) for 72 hrs. Cell-free controls (CFCs) were included to validate cell-secreted products. (**B**) Densitometric analysis of Western blots pertaining to samples collected from MSCs. Bands from MMP-1, MMP-3, CCL-2, and IL-6 blots were quantified by using ImageJ and normalized to corresponding MMP-2 bands. Results are from three experimental replicates. #, p < 0.05, compared with vehicle control; *, p < 0.05.

To investigate the effects of MPs on MSC cytokine secretion, MSCs were treated with EC- or CC-derived MPs or Sup (25 μg protein/ml) for 72 hrs and CM was collected and analysed by MMP-1, MMP-3, CCL-2/MCP-1 and IL-6 Western blots (Fig. 5A). Blots of MMP-2, an MSC-secreted factor that does not respond to MPs or Sup, were included as loading controls in Western blots and used for normalization in densitometric analyses (Fig. 5B). CFCs consisting of identically treated cell-free tissue culture plastic (TCP) were also included. CC MPs increased MSC secretion of MMP-1, MMP-3, CCL-2/MCP-1 and IL-6 compared with vehicle control. EC MPs also increased MSC secretion of MMP-1, MMP-3, CCL-2/MCP-1 and IL-6 (Fig. S6), but only CCL-2/MCP-1 was induced at levels comparable to those stimulated by CC MPs. CC and EC Sup both increased MSC secretion of MMP-1, MMP-3, CCL-2/MCP-1 and IL-6. CC Sup was significantly more potent in its increases compared with EC Sup, particularly in the case of IL-6. None of the molecules analysed was present in any of the CFC controls, indicating that whatever changes seen in MSC samples were because of cell-secreted molecules and not because of molecules merely being added as part of the treatments (MMP-1 and CCL-2 were detected in EC CM collected after 3 days. However, the EC-derived products used as treatments in this experiment were collected after 24 hrs, and levels of these proteins were undetectable in CFC control samples.)

These experiments were repeated with ECs and CCs treated with CC- and EC-derived MPs and Sup, respectively, to determine whether MP effects on cytokine secretion differed with the receiving cells (Fig. 5A). CC Sup increased EC secretion of MMP-1, MMP-3, CCL-2/MCP-1 and IL-6 compared with vehicle controls. EC Sup increased CC secretion of MMP-1. ECs were unresponsive to EC Sup, and CCs were unresponsive to CC sup (data not shown). Thus, both ECs and CCs were responsive to non-MP factors secreted by each other, but not to their own. Interestingly, neither ECs nor CCs responded to MPs derived from either cell type. Thus, MSCs appear to be particularly responsive to MPs.

### MPs activate NF-κB signalling in MSCs

**Fig. 6:**
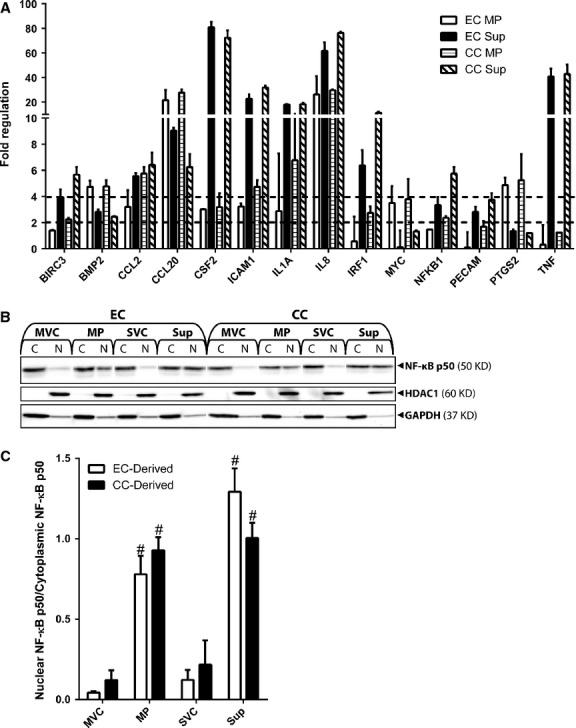
Microparticles (MPs) and Sup activate mesenchymal stem cell (MSC) NF-κB signalling. (**A**) MSCs treated with endothelial cell (EC) or CC MPs or Sup (100 μg protein/ml) were analysed with Signal Transduction PathwayFinder PCR Arrays, which measures changes in known transcription targets of cellular signalling cascades. Fold changes of 0 represent vehicle control values. Dashed lines mark fold changes of 2 and 4. (**B**) MSCs were treated with EC or CC MVC, MP, SVC or Sup for 1 hr, separated into cytoplasmic (C) and nuclear (N) fractions, and analysed by NF-κB p50, HDAC1 and GAPDH Western blots. (**C**) Densitometric analysis of Western blots data. NF-κB p50 bands were quantified, and nuclear values were normalized to corresponding cytoplasmic values. Results are from three experimental replicates. #, p < 0.05, compared with vehicle control.

The following experiments were aimed at identifying the MSC signalling pathways activated by EC and CC MPs under control conditions. To identify likely candidate signalling pathways activated by MPs in MSCs, cultures of MSCs treated with EC or CC MPs for 4 hrs were analysed with Signal Transduction PathwayFinder PCR Arrays (Fig. 6A). These arrays probe for expression of known transcription targets of various signalling pathways, including the Wnt, hedgehog, TGF-β, PI3 kinase/AKT, Jak/SRC, p53, NFAT, CREB, Jak-Stat, PKC, phospholipase C, LDL and NF-κB pathways. Treatment with MPs and/or Sup induced increased MSC expression of several transcription targets compared with vehicle controls, including BIRC3, BMP2, CCL2, CCL20, CSF2, ICAM1, IL1A, IL8, IRF1, MYC, NFKB1, PECAM, PTGS2 and TNF. The majority of these genes, BIRC3, CCL20, ICAM1, IL1A, IL8, NFKB1, PECAM and TNF, are transcriptional targets of the NF-κB pathway, identifying NF-κB as a likely candidate signalling cascade activated by Sup and MPs. To validate MSC NF-κB activation, MSCs treated with EC or CC MPs or Sup for 1 hr were separated into cytoplasmic and nuclear protein fractions and analysed by NF-κB p50 Western blots (Fig. 6B). Cytoplasmic/nuclear protein fractionation was validated with HDAC1 and GAPDH Western blots, which showed HDAC1 and GAPDH localization to nuclear and cytoplasmic fractions, respectively. Densitometric analysis of Western blot data confirmed significant increases in the ratio of nuclear to cytoplasmic NF-κB p50 in response to MP and Sup stimulation compared with vehicle controls (Fig. 6C). Thus, treatment with EC and CC MP and Sup caused significant increases in nuclear NF-κB p50 compared with vehicle controls, which agreed with PCR array results and suggested activation of the NF-κB signalling pathway in MSCs by EC- and CC-secreted factors, including MPs.

## Discussion

Intercellular interactions have traditionally been thought to include cell–cell, cell–matrix and cell-soluble factor interactions. However, a fourth avenue of crosstalk involving MPs has proven to occupy a pivotal role in intercellular communication [54]. MPs are selectively packaged with specific biomolecules, including protein, mRNA and microRNA, as they are released by parent cells [54–56]. As stable membrane enclosed vesicles, MPs are able to target to remote tissues to effect traditional pathways of intercellular interaction. For example, MPs contain membrane proteins, including cadherins and other cell adhesion molecules, which, upon binding to receptors on the receiving cell, activate signalling pathways and cellular responses as if the parent and recipient cells were in direct physical contact [57]. Matrix receptors on the surfaces of MPs also localize ECM molecules to the membranes of receiving cells, effectively facilitating cell–matrix interactions [37]. Finally, MPs release growth factors and cytokines as they degrade, regulating their delivery to target cells [39–43]. However, MPs are also able to signal via intercellular transference of biomolecules, which occurs when the membrane of the MP fuses with that of the receiving cell. Once incorporated by receiving cells, the transferred MP biomolecules may elicit biological changes [32,35–38,58]. Thus, MPs are optimally suited for their roles as a compact signalling platform, forming stimulatory complexes with receiving cell surface receptors and transferring biomolecules to the membranes and interiors of recipient cells.

Here, we investigated the role of MPs and non-MP secreted factors (Sup) in signalling among MSCs, ECs and CC, cell types known to interact under both physiological and pathological conditions *in vitro* and *in vivo* [5,9–12,59,60], observing key cell responses in MSCs when treated with Sup and MPs. (Note: Interestingly, in our hands, MSCs did not produce detectable levels of MPs, despite several previously published reports to the contrary. We believe that these discrepancies are because of MSC biological state and source, as many of the studies that reported on MSC MPs used commercially available MSCs [61,62], whereas the study presented here used low passage, freshly isolated primary cells.) First, we showed that MPs transfer both membrane and cytosolic molecules from ECs to MSCs in a process that requires packaging of biomolecules within MPs. Only MP-associated molecules are taken up and retained by MSCs, suggesting that EC MPs are necessary and sufficient for transferring membrane and cytosolic components between ECs and MSCs.

Microparticle-mediated signalling between MSCs and ECs independent of biomolecular transference was also observed. First, MPs were shown to be particularly chemoattractive to MSCs, and MPs isolated from a fibrosarcoma cell line were significantly more chemoattractive to MSCs than EC MPs. Given that increased levels of MPs are released at sites of injury/cancer and in response to cytokine stress [25,63–66], and that MSCs home to sites of injury and cancer [9,10,67–69], these results suggest a role for MPs in attracting MSCs and other cells to sites of injury/cancer. These results also suggest that the identity of the parent cells of MPs affect the migration response of the receiving cells.

Analysis of the effects of MPs on MSC cytokine secretion revealed that both Sup and MPs activate secretion of MMP-1, MMP-3, CCL-2 and IL-6. Cytokines such as these are responsible for many of the trophic effects reported for MSCs. For example, MSC-secreted IL-6 has been shown to maintain MSC stemness [70], promote adenocarcinoma tumour growth [71], delay neutrophil apoptosis and inhibit progenitor cell differentiation into dendritic cells by impairing their antigen-presenting function [72–78]. CCL-2/MCP-1 regulates MSC homing to breast cancer tumours [10]. MSC-secreted MMP-3 has been linked to the pro-angiogenic effects of MSCs [79]. MMP-1 is secreted by MSCs as they migrate towards tumours [80], and having reported here that MPs induce both chemotaxis and MMP-1 expression in MSC, it appears that MPs are particularly supportive of MSC migration. As in the migration studies, MP effects on MSC cytokine secretion differed with the identity of the parent cells, as CC MPs proved to be significantly more inductive than EC MPs. On the other hand, both EC and CC MPs activate MSC NF-κB signalling at comparable levels, suggesting that the particular potency of CC MPs may be grounded in properties unrelated to this particular intracellular signalling cascade. Furthermore, MP effects on cytokine secretion were heavily dependent on the identity of the receiving cell. In fact, of the three cell types tested, MSCs were the only cell type that responded to MP treatment in terms of the tested cytokines. Thus, MSCs may be particularly responsive to MP signalling, and we are currently conducting studies aimed at determining the mechanisms underlying the abilities of EC and CC MPs to specifically target MSCs.

In summary, we have demonstrated signalling between MSCs and MPs *in vitro*. Our findings that MPs activate MSC migration and cytokine secretion have potential applications towards elucidating the mechanisms underlying MSC homing to and modulation of injury and cancer sites. Our studies have also revealed that the identity of the parent and target cells affects how MP signals are received and interpreted, indicating an exciting and complex role of MPs in transferring disease state information between cells. Thus, this study has revealed unexpected layers of sophistication regulating MP-mediated signalling between ECs and MSCs and provides evidence towards establishing MPs among the functional communication channels of EC-MSC crosstalk.

## Supporting information

**Figure S1** MSCs were analysed via flow cytometry for expression of CD73, CD90, CD105, CD45, CD19, CD34 and CD14.

**Figure S2** EC supplements are required to maintain EC viability during 24 hrs of SF culture.

**Figure S3** Ectosome and exosome fractions were isolated using differential centrifugation and each were tested for their effects on MSC (A) migration and (B) proliferation.

**Figure S4** Western blot analysis for GFP (and GAPH) of MP and Sup media fractions isolated via ExoQuick-TC (EQ) or ultracentrifugation (UC).

**Figure S5** Western blot analysis for organelle markers of protein samples (10 μg total protein) from parent cells (Cell) and MP and Sup media fractions isolated from ECs and CCs.

**Figure S6** EC MP induced MSC cytokine secretion.
