# Supplementary figures and images for: Endothelial and cancer cells interact with mesenchymal stem cells via both microparticles and secreted factors
Source: J Cell Mol Med. 2014 Sep 23;18(12):2372–84. doi: 10.1111/jcmm.12391 (PMC4302643; doi:10.1111/jcmm.12391)

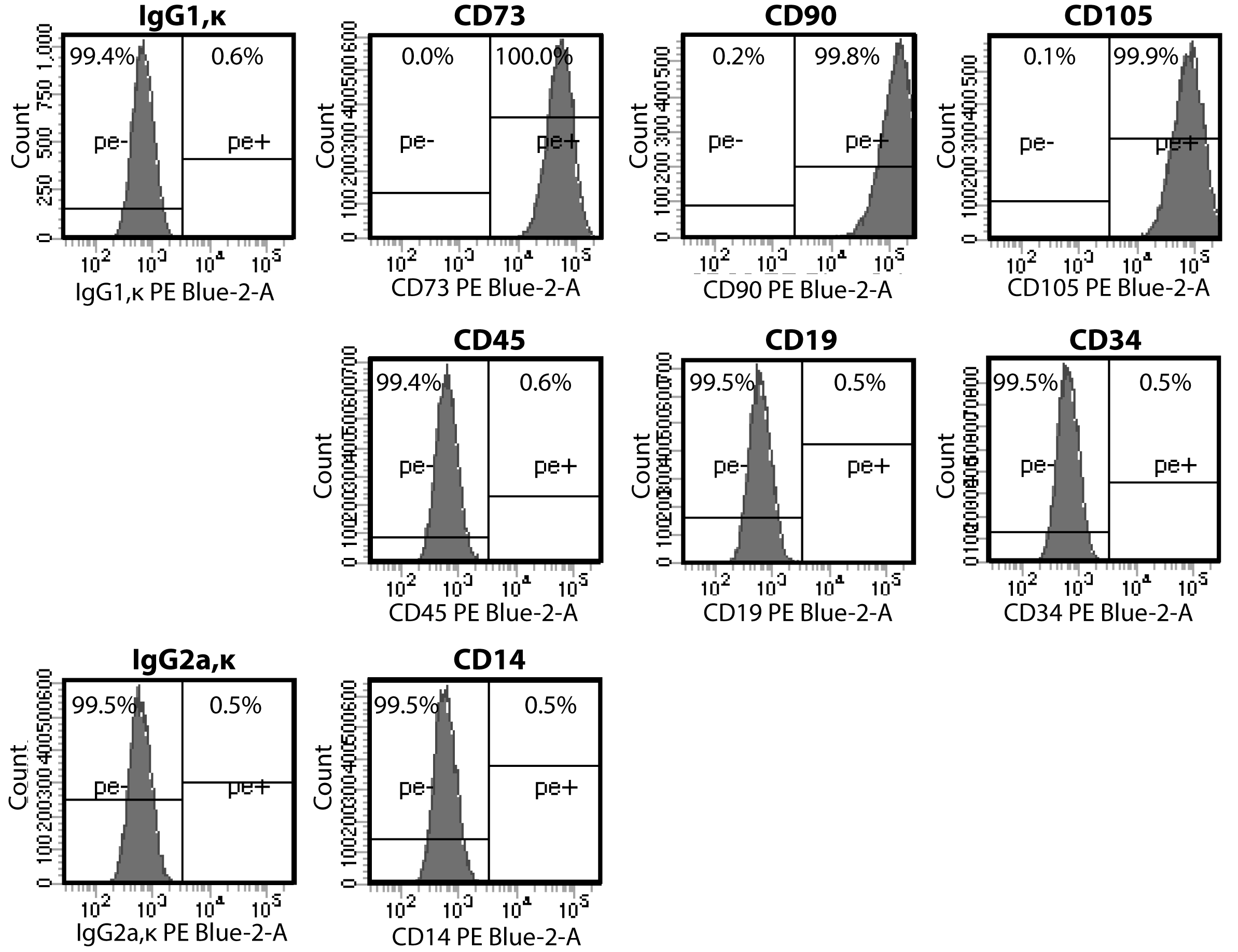

Supplement: Supplementary file 1 — Figure S1 MSCs were analysed via flow cytometry for expression of CD73, CD90, CD105, CD45, CD19, CD34 and CD14. [file jcmm0018-2372-sd1.tif]

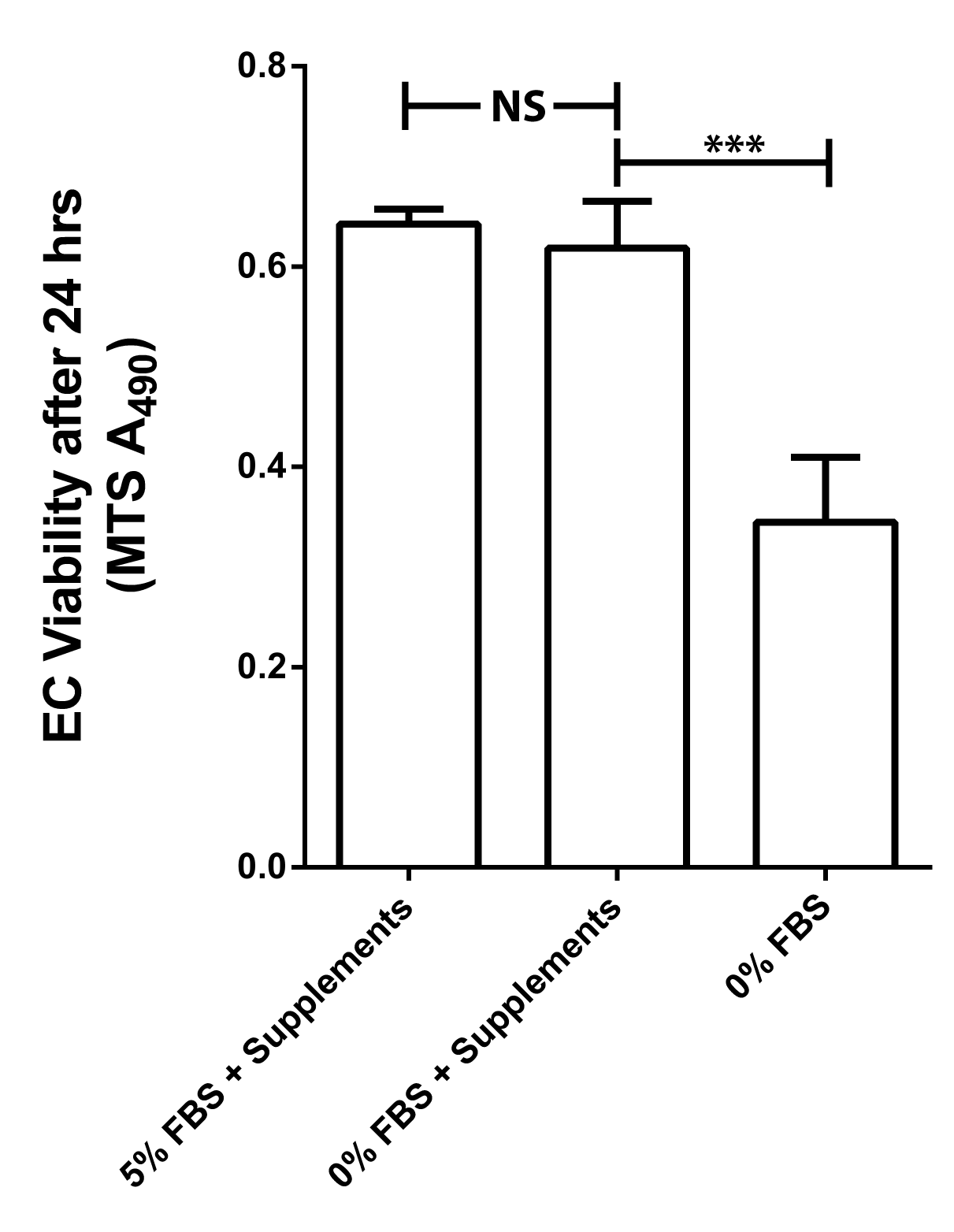

Supplement: Supplementary file 2 — Figure S2 EC supplements are required to maintain EC viability during 24 hrs of SF culture. [file jcmm0018-2372-sd2.tif]

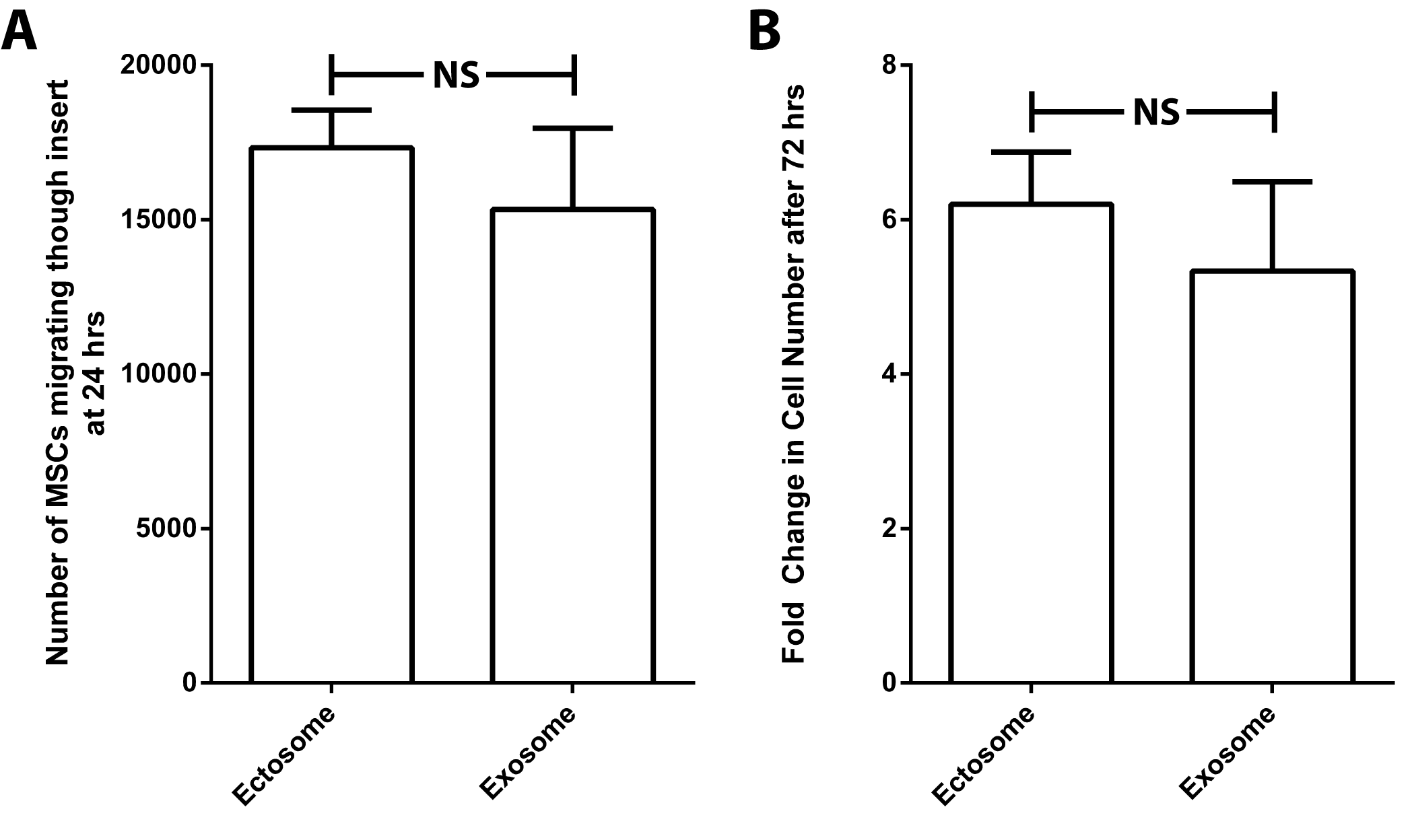

Supplement: Supplementary file 3 — Figure S3 Ectosome and exosome fractions were isolated using differential centrifugation and each were tested for their effects on MSC (A) migration and (B) proliferation. [file jcmm0018-2372-sd3.tif]

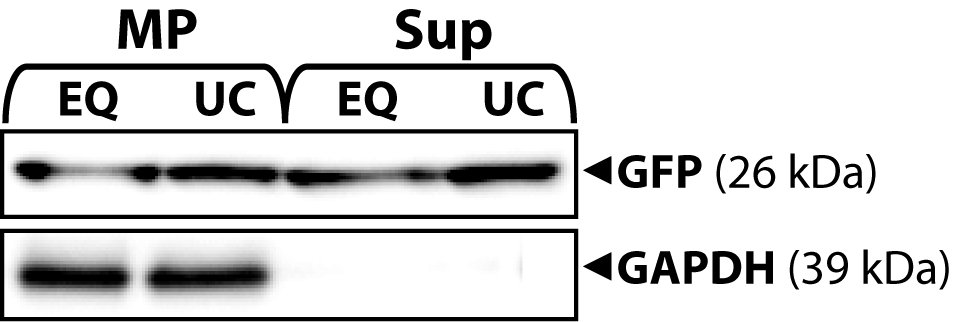

Supplement: Supplementary file 4 — Figure S4 Western blot analysis for GFP (and GAPH) of MP and Sup media fractions isolated via ExoQuick-TC (EQ) or ultracentrifugation (UC). [file jcmm0018-2372-sd4.tif]

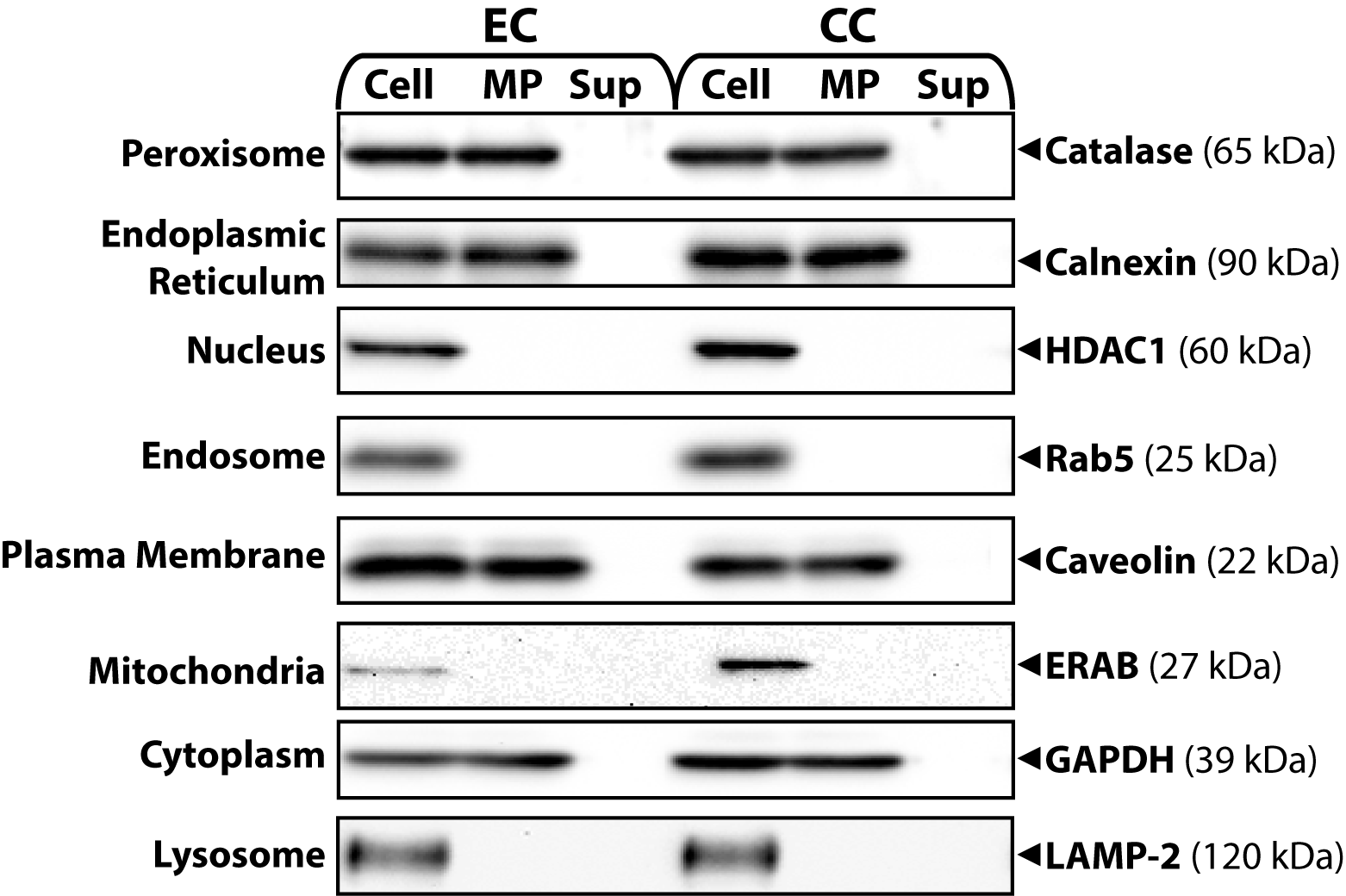

Supplement: Supplementary file 5 — Figure S5 Western blot analysis for organelle markers of protein samples (10 μg total protein) from parent cells (Cell) and MP and Sup media fractions isolated from ECs and CCs. [file jcmm0018-2372-sd5.tif]

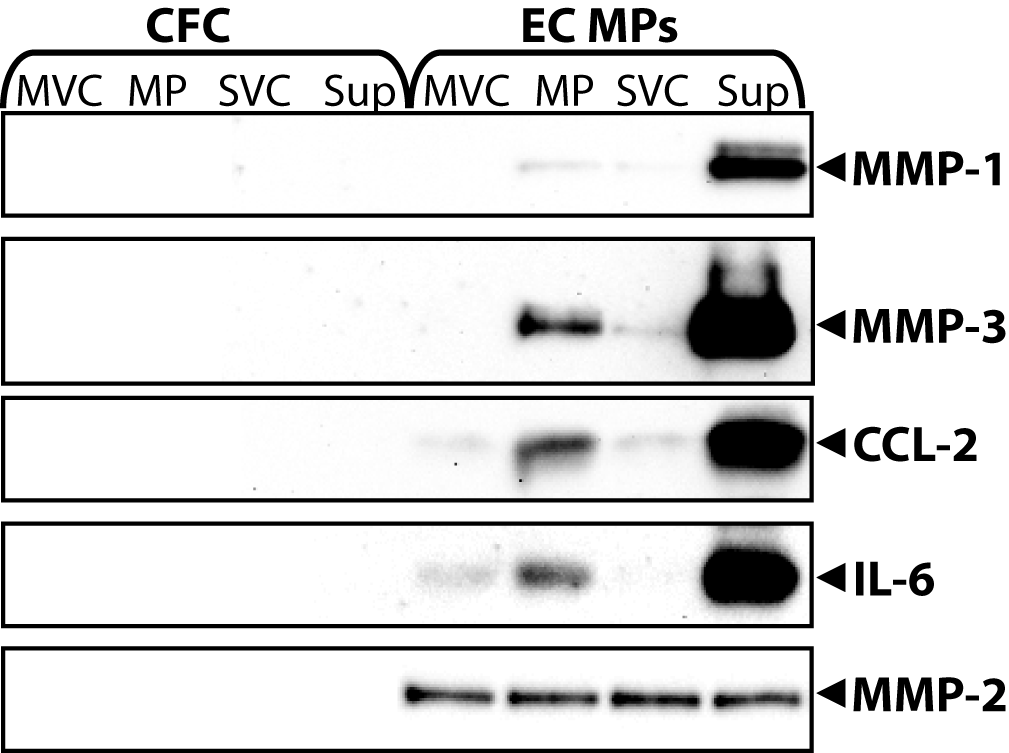

Supplement: Supplementary file 6 — Figure S6 EC MP induced MSC cytokine secretion. [file jcmm0018-2372-sd6.tif]
